# The Missing Incisors in Man, Which Are They?

**Published:** 1885-04

**Authors:** Andrew Wilson


ARTICLE VII.

### THE MISSING INCISORS IN MAN—WHICH ARE THEY?

BY ANDREW WILSON, L. D. S.

The number of incisor teeth in what is called the typical dentition in placental mammalia being in each jaw six, or three pair (one central and two lateral), while that in man is normally two pair (one central and one lateral), the question I propose raising is, which of the lateral pairs has been suppressed? The answer given by the authorities on Dental Anatomy is, the outermost, *i. e.*, the third on each side. Mr. C. S. Tomes, in his manual, second edition, page 9, says:—"The human subject does not possess the third incisor . . . . so that a somewhat abrupt change of form in passing from the incisors to the canines . . . . is no more than might be anticipated." And at page 286 he gives as a general rule, "It is usually said that when incisors are missing from the full typical number, they are lost from the outer end of the series, that is to say, if there is but one incisor, it is $I^1$; if two, $I^1$ and $I^2$. There are many exceptions to this, *e. g.*, the first incisor is the first to disapper in the otter (?), walrus, and some others."

The first named is evidently a misprint for the Otariæ (eared seals), the number of incisors present being very varied in the different genera among the Phocidæ.

As in the superior maxilla we have the advantage of the incisor teeth being restricted to a well-defined portion, the intermaxillary, originally a distinct bone, I will, in what follows, speak of the upper incisors only.

Cases in which we find the full typical number of incisors present and of normal form in man are seemingly very rare, but cases in which, while only the normal number was present on one side, there were three on the other, are much more frequent.

These extra teeth usually follow the lateral type, but we have a model in the museum of our Society of one following the central type.

Of the first class I have only met with two (exclusive of one* in the temporary dentition), as against seven of the second class, and as in these (with two doubtful exceptions) the lateral second from the canine seemed to me the intercalated one, I have been in the habit of teaching that I considered the suppressed incisor in man to be the second, not the third, the authority of the manual notwithstanding.

This view is very materially strengthened if we include as incisors those abnormally formed teeth which so frequently show themselves in the intermaxillary portion of maxillary bones.

These almost invariably take up a position either between the normal lateral and the central incisor, or the mesial line, most frequently within the dental arch.

Looking to the forms of these teeth, we may arrange them in two groups, the first—by far the more common—are pointed conical teeth (the typical supernumerary tooth); the second are usually much larger, with very irregularly formed cutting edges, frequently broad and multi-tuberculated.

The first group I hold to be lateral incisors taking on the rudimentary form, which we find, as a rule, to precede

---

*In this the teeth formed a perfect arch, and the central and second incisors were geminated, each having its own pulp cavity. The after history of this case would have been of considerable value, but the accidental death of the boy made it only partial. A second model shows that the second incisor on the right side was succeeded by a permanent one, which erupted *outside* the arch. His father (a medical lecturer) reported that a lateral had appeared on the other side in succession to the *third* temporary incisor, and that there was no appearanee of a second. Still, sufficient time had not elapsed to say it might not have comc; at the same time, if it had done so, it would have been out of the arch.

suppression, and which we now find not unfrequently in undoubted lateral incisors.

I may remark in passing that it is from the more frequent appearance of this form in the normal lateral, coupled with the seemingly now more frequent suppression of this tooth, that we conclude that in man there will in course of time be only the central incisors left.

Those of the second group I regard as, when large, malformed incisors of the central type, and when smaller, of the lateral; and I think a close inspection of these forms will bear me out.

Holding these opinions, I was much pleased to learn, through a paper by Professor Turner on "The Relation of the Alveolar Form of Cleft Palate to the Incisor Teeth and the Intermaxillary Bones," read before the Royal Society here in December last, that Dr. Paul Albrecht had, while investigating the anatomy of cleft palate, been led to the same decision regarding the suppressed incisors in man.

For the benefit of those members who may not have an opportunity of seeing that paper, which strongly supports Dr. Albrecht's views, I will, in conclusion, very briefly give the chief points which led Dr. Albrecht and Professor Turner to that decision.

In very much the larger number of cleft palate cases examined by them they found that the alveolar fissure, in place of being in the line of the maxillo-intermaxillary suture, that is, between the outer incisor and the canine, was to the *mesial* side of the outer incisor and so in the body of the intermaxillary bone, the maxillo-intermaxillary suture coexisting with it. Minute examinations of a large series of superior maxillæ led to the detection of traces more or less decided of an "intra-incisive" suture (in the plane of which the cleft usually occurs), and to the conclusion that originally the intermaxillary portion consisted of two bones on each side, the mesial one of which, in man, carried the socket of the central incisor, the other that of the lateral.

In those cases of cleft palate in which an extra incisor was present, its sockets was *invariably in the mesial portion* along with that of the central, showing it to be the second incisor of the typical dentition.—*London Dental Record.*

